# *Salmonella* Establishment in Agricultural Soil and Colonization of Crop Plants Depend on Soil Type and Plant Species

**DOI:** 10.3389/fmicb.2019.00967

**Published:** 2019-05-15

**Authors:** Sven Jechalke, Jasper Schierstaedt, Marlies Becker, Burkhardt Flemer, Rita Grosch, Kornelia Smalla, Adam Schikora

**Affiliations:** ^1^Institute for Phytopathology, Justus Liebig University Giessen, Gießen, Germany; ^2^Leibniz Institute of Vegetable and Ornamental Crops, Plant-Microbe Systems, Großbeeren, Germany; ^3^Federal Research Centre for Cultivated Plants, Julius Kühn-Institut (JKI), Institute for Epidemiology and Pathogen Diagnostics, Braunschweig, Germany

**Keywords:** internalization, plant defense, *Salmonella*, persistence, soil, crop plants

## Abstract

Human pathogenic bacteria, such as *Salmonella enterica*, are able to colonize crop plants. So far, not much is known about biotic and abiotic factors influencing this colonization in field soil. This understanding, however, is imperative for the provision of safe fresh produce to the consumer. In this study, we investigated the effects of soil type, organic fertilization, plant species and the way of *Salmonella* entry into the plant production system, on the survival of *S. enterica* in soil as well as the colonization of plants. The selected *S. enterica* serovar Typhimurium strain 14028s, *S.* Typhimurium strain LT2 and *S.* Senftenberg were able to persist in soil for several weeks. *Salmonella’s* persistence in soil was prolonged in loamy, if compared to sandy soil, and when applied together with organic fertilizer. The leaves of lettuce and corn salad were colonized by *S. enterica* providing evidence for internalization from the soil *via* the root. Colonization rates were affected by soil type, plant species and *S. enterica* strain. Overall, *S. enterica* was detected in leaves of 0.5–0.9% of the plants, while lettuce was more frequently colonized than corn salad. Plants grown in sandy soil were more often colonized than plants grown in loamy soil. After spray inoculation, *S. enterica* could be detected on and in leaves for several weeks by cultivation-depending methods, confirmed by confocal microscopy using GFP-labeled *S.* Typhimurium 14028s. On the one hand, transcriptome data from *S.* Typhimurium 14028s assessed in response to lettuce medium or lettuce root exudates showed an upregulation of genes associated with biofilm formation and virulence. On the other hand, lettuce inoculated with *S.* Typhimurium 14028s showed a strong upregulation of genes associated with plant immune response and genes related to stress response. In summary, these results showed that organic fertilizers can increase the persistence of *Salmonella* in soil and that soil type and plant species play a crucial role in the interactions between human pathogens and crop plants. This understanding is therefore a starting point for new strategies to provide safe food for the consumer.

## Introduction

The number of disease outbreaks, associated with fresh food of non-animal origin contaminated with human pathogens, increased in the EU from 2008 to 2011. Among them, the top ranked pathogen-food associations were raw eaten leafy greens contaminated with *Salmonella* ssp. (EFSA, 2013). In 2016, 0.21% of the 2,429 vegetable samples were positively tested for different *Salmonella* serovars, most of them at the retail stage (EFSA, 2017b). More recently, the *S. enterica* serovar Agona outbreak in several EU countries in 2018 was linked to ready-to-eat products containing cucumbers (EFSA, 2018). One potential source of contamination is the soil on which the plant is growing. Although the risk of plant colonization by human pathogens from soil is lower than, e.g., in hydroponic solution (Hirneisen et al., 2012), internalized *Salmonella* would be protected against post-harvest processing treatments and might therefore pose a risk for human health. Today, the pre-harvest colonization of leafy greens by *Salmonella* and factors influencing the extent of this contamination are not fully understood.

Potential sources of soil contamination with human pathogens are manure, used as organic fertilizer, and contaminated irrigation water (Beuchat, 2002). Pigs are typical hosts of *Salmonella*. Between 2006 and 2007, about 10.3% of the slaughter pigs in the EU were *Salmonella*-positive (EFSA, 2008). *S. enterica* serovar Typhiumurium (*S*. Typhimurium) was the most commonly reported serovar in pig herds and the second top-ranked serovar isolated from pork (EFSA, 2017b). Poultry are other typical hosts of *Salmonella*. For example, in 2017 a multi-country outbreak of *S.* Enteritidis was linked to eggs from Poland (EFSA, 2017a). Therefore, *Salmonella* can reach agricultural soils *via* fertilization with pig or poultry manure. Previous reports showed that *Salmonella* persist in soil for at least 21 days when originating from pig manure (Pornsukarom and Thakur, 2016) and up to one year when originating from poultry manure (Hruby et al., 2018), indicating that soils can act as a long-term *Salmonella* reservoir. Factors influencing the survival of *S. enterica* in manure and manure-amended soil were reviewed in Ongeng et al. (2015) and include the physical and chemical characteristics of both manure and soil, and also weather and atmospheric conditions, biological interactions, agricultural and livestock management practices, strain variation and physiological age of the cells.

Once *Salmonella* is in the soil, the most probable routes of contamination of crop plants are *via* the rhizosphere and root and *via* soil splashing on leaves, flowers or fruits during overhead irrigation or rainfall. All might result in internalization of *Salmonella* into the edible plant parts, as reviewed by Hirneisen et al. (2012). For example, even though the numbers of *S. enterica* internalized in lettuce were reported to decrease over time of cultivation, bacteria persisted in plant tissues (Jablasone et al., 2005). The observed negative correlation between internal colonization and plant mass (Franz et al., 2007), could indicate an infection rather than a contamination or colonization and point to a potential disadvantage for the host plant.

Nonetheless, not much is known about factors influencing the colonization of plants by *S. enterica* directly or indirectly such as soil type, organic fertilizer application, plant species, or *Salmonella* strain. Furthermore, reports on the internalization of *Salmonella* into lettuce plants were not always consistent, or reported only low numbers of cases (Honjoh et al., 2014). This supports the notion that internalization rates are rather low and/or depend on certain experimental conditions. One possible explanation for the low internalization rate of human pathogens into plant tissues might be the fact that *Salmonella* triggers a plant immune response, which might, to a certain level, prevent the colonization.

In this study, we investigated the colonization of leafy greens *via* soil inoculated with *S.* Typhimurium strains 14028s and LT2 as well as *S.* Senftenberg. Furthermore, in microcosm experiments we assessed the potential of different factors to influence the persistence of *Salmonella* in soil. These factors included soil texture, application of organic fertilizers (pig manure, chicken litter), *Salmonella* strain and manner of *Salmonella* application. We hypothesized that those factors could influence the persistence of *Salmonella* in soil and could also directly or indirectly influence the colonization rate of crop plants. In parallel, adaptations of *S. enterica* to the plant environment as well as the plant immune response to *Salmonella* inoculation were investigated on transcriptome level and the potential internalization routes were explored using confocal laser scanning microscopy (CLSM) and GFP-labeled *S.* Typhimurium 14028s. Our results show that the persistence of *Salmonella* in soil is enhanced in loamy soil, if compared to sandy soil and by an application of organic fertilizer, however, only when *S. enterica* was introduced into the plant production system together with the fertilizer. The colonization rates of lettuce and corn salad were relatively low and varied substantially between the soil types, plant species and *Salmonella* strains. CLSM confirmed secondary root emerging zones, root hairs, stomata and hydathodes as potential internalization sites of *Salmonella*. The transcriptome analyses showed that *S.* Typhimurium 14028s responded differently to lettuce medium and lettuce root exudates, indicating an active adaptation process. Furthermore, the lettuce transcriptome revealed that *S.* Typhimurium 14028s triggered a plant immune response, supporting the hypothesis that both organisms, *Salmonella* and the colonized plant, play an active role during the colonization process.

## Materials and Methods

### *Salmonella* Strains Used in This Study

*Salmonella enterica* serovar Senftenberg (*S*. Senftenberg), obtained from Nicola Holden, The James Hutton Institute, Dundee, and John Coia, SSSCDRL, Glasgow (Scotland, United Kingdom) (Elviss et al., 2009), *S. enterica* serovar Typhimurium 14028s (*S.* Typhimurium 14028s) ATCC 14028, obtained from Dr. Isabelle Virlogeux-Payant, INRA Tours (France) and *S. enterica* serovar Typhimurium strain LT2 (*S.* Typhimurium LT2) DSM 18522 (Jarvik et al., 2010) were used in this study. Rifampicin resistant mutants were obtained by overnight culture on plates with LB agar (Carl Roth GmbH + Co. KG, Karlsruhe, Germany) containing rifampicin (50 mg/L, SERVA, Heidelberg, Germany) as described previously (Fornefeld et al., 2017).

To visualize the colonization patterns in lettuce (*Lactuca sativa* L. cultivar Tizian) and corn salad (*Valerianella locusta* L. cultivar Verte à coeur plein 2), *S.* Typhimurium 14028s was GFP-labeled with the plasmid pSM1890 (Haagensen et al., 2002). To this end, a triparental mating was performed between *S.* Typhimurium 14028s, *Escherichia coli* carrying the *gfp*-tagged IncQ plasmid pSM1890 GFP derived from the IncQ plasmid pIE723 (Richter and Smalla, 2007), and *E. coli* carrying the helper plasmid R751 (Thorsted et al., 1998). The strains were streaked on LB plates containing the respective antibiotics, incubated overnight at 37°C. Cells were harvested with a loop and resuspended in 1 mL of 10 mM MgCl_2_. Cell solutions were combined and mixed, the suspension was centrifuged (4000 × *g*, 3 min) and the supernatant was removed. Cells were resuspended in the residual liquid and placed on a filter disc (0.22 μm, Durapore^®^ membrane filters, Merck, Darmstadt, Germany) on LB and incubated overnight at 28°C. The following day, cells were resuspended from the filter by vortexing in 5 mL of 10 mM MgCl_2_. Transconjugants were selected by plating of serial dilutions on LB plates containing rifampicin 50 mg/L, gentamicin 10 mg/L (Carl Roth GmbH + Co. KG) and streptomycin 50 mg/L (Carl Roth GmbH + Co. KG) and incubation at 28°C. After 24 h, green fluorescent colonies were picked and plated on XLD agar (Carl Roth GmbH + Co. KG), subsequently incubated for 24 h at 37°C to confirm the presence of *Salmonella*, indicated by the formation of black colonies.

### Origin and Characteristics of Agricultural Soils, Manure, and Chicken Litter

Two types of agricultural soil were chosen for the experiments, a sandy and a loamy soil. The sandy soil was characterized as Arenic-Luvisol with less silty sand and 5.5% clay, pH 6.1 and organic carbon content of 0.9% (diluvial sand, DS) and the loamy soil as Gleyic-Fluvisol with heavy sandy loam and 27.5% clay, pH 6.7 and organic carbon content of 1.8% (alluvial loam, AL) (Rühlmann and Ruppel, 2005; Schreiter et al., 2014).

Pig manure used in this study was obtained from an experimental pig husbandry farm at the Friedrich-Loeffler-Institute in Braunschweig, Germany. Chicken litter was obtained from a free-range chicken stable in Lower Saxony, Germany. Pig manure and chicken litter were stored at 4°C until the start of the experiment. Both fertilizers were tested negatively for the presence of *Salmonella* before the experiments. The matrix characterization of the organic fertilizers was performed by LUFA Nord-West (Hameln, Germany) and results are given in Supplementary Table S1.

### Design of the Experiment

A greenhouse microcosm experiment was performed to investigate the influence of organic fertilizer application (pig manure and chicken litter), *Salmonella* strain and manner of *Salmonella* application on the persistence of *Salmonella* in agricultural soils (DS and AL soil) as well as on the colonization of plants (lettuce and corn salad).

For the fertilizer treatments, soils were mixed with organic fertilizers to obtain a maximum N application of approximately 170 kg N/ha. Therefore, the N content of the organic fertilizers was determined (see above). The amount of fertilizer was calculated considering a depth of incorporation of 5 cm into the soils and equals 0.23 and 0.26 g N/kg DS and AL soil, respectively, due to different densities of the two soils. The amount of 170 kg N/ha and year is the maximum amount allowed to be applied on agricultural fields in Germany (Bundesministerium der Justiz und für Verbraucherschutz, 2017). Water was added to the fertilizer treatment and the control soil without fertilizer to obtain a maximum water holding capacity (WHC_max_) of 50%.

About 28–44 days after the amendment with organic fertilizers (mixing day), lettuce and corn salad were transferred at a 2–3 leaf stage to the soil treatments in 100 mL polystyrene flower pots. In the following, this day is referred to as day zero. Special care was taken during the transfer to avoid contamination of the leaves with soil particles. The plants were pre-grown in planting trays containing a mixture of 1:1 (vol/vol) DS or AL soil mixed with bedding substrate (Substrat 1, Klasmann-Deilmann GmbH, Geeste, Germany) to support the germination and growth of the seedlings.

*Salmonella* strains were mixed into the soil either together with the organic fertilizers (on mixing day) to simulate a contamination by fertilization (“stored” treatment) or directly before the transfer of the plants (on day 0), simulating the contamination by irrigation water (“fresh” treatment). Therefore, *Salmonella* strains were first streaked on lettuce medium (LM) agar plates which were freshly prepared as described previously (Fornefeld et al., 2017) containing 50 mg/L rifampicin. After overnight incubation at 28°C, *Salmonella* strains were harvested from agar plates, resuspended in 10 mM MgCl_2_ solution and the number of bacterial cells was estimated by the measurement of the optical density (OD) at 600 nm. All strains were inoculated at final cell number of 10^6^ colony forming units (CFU) per g dry soil.

The pots were watered from below, as needed, and incubated in the greenhouse at 20°C, 16 h light.

### Quantification of *Salmonella* in Soil by Plate Counts

On mixing day, as well as during the incubation period (on days 4, 7, 21, 35, or 49 after planting), soils of 4 replicate pots per treatment were mixed thoroughly and from each pot 1 g of soil was transferred to 50 mL screw cap centrifugation tubes (SARSTEDT AG & Co. KG, Nümbrecht, Germany), 9 mL of 10 mM MgCl_2_ solution was added and the soil slurry was mixed by vortexing for 1 min. MgCl_2_ was used in order to allow comparisons to previous experiments. Duplicates of serial dilutions were dropped (10 μL) in two technical replicates on XLD agar containing rifampicin 50 mg/L. CFU were enumerated after an overnight incubation at 37°C. Additionally, 100 μL and 1000 μL were spread on XLD agar (Carl Roth GmbH + Co. KG) when low cell counts of *Salmonella* were expected.

### Quantification of Phyllosphere Colonization

In order to investigate the relation between persistence of *Salmonella* in soil and colonization of crop plants, lettuce and corn salad leaves were sampled during the growth period, in parallel to the soil sampling (on days 4, 7, 21, 35, or 49 after planting). Only leaves that were not in contact with the soil were sampled. Leaves were sampled using sterilized scissors and forceps and two plants were pooled into one single 50 mL screw cap centrifugation tube. Afterwards, the leaves were shredded with sterilized scissors and 10 mL Buffered Peptone Water (BPW, Carl Roth GmbH + Co. KG) was added. Following the overnight incubation at 37°C under shaking conditions (140 rpm), 10 μL of the suspension was transferred to 190 μL Rappaport Vassiliadis Broth (RVS, Carl Roth GmbH + Co. KG) in 96-well plates and incubated overnight at 42°C. Ten microliter of both enrichments (BPW and RVS) were dropped on XLD plates containing rifampicin (50 mg/L) and incubated overnight at 37°C to confirm the presence of *Salmonella*.

### Statistical Analysis

For *Salmonella* CFU counts in soil, slopes of linear regressions were analyzed by a generalized linear model using the mixed procedure (SAS 9.4; SAS institute Inc., Cary, NC, United States). Tukey tests were performed with the procedure glimmix of SAS. Plant colonization in dependency of time after the transfer of the plants was analyzed by a linear model using the lm function (R; version 3.2.1). Differences in numbers of colonized plants between treatments were analyzed by Pearson’s Chi-squared test for count data using the chisq.test function. Results were considered significant when *p* < 0.05.

### *Salmonella* Persistence in the Phyllosphere

To investigate the ability of *Salmonella* to persist on and in lettuce and corn salad leaves, the plants were spray-inoculated with different cell counts of *S.* Typhimurium 14028s and over time, the presence and quantity of *Salmonella* was determined by enrichment and direct CFU count approaches. The rifampicin resistant *S.* Typhimurium 14028s strain was pre-grown on LB plates containing rifampicin (50 mg/L) overnight at 37°C. Bacterial cells were harvested and resuspended in 10 mM MgCL_2_ containing Tween 20 (0.02%, SERVA). Lettuce and corn salad plants at 5–10 leaf stage, which were pre-grown in potting soil, were sprayed with *S.* Typhimurium 14028s suspensions in cell counts between 10^7^ and 10^2^ CFU/mL. Spray-inoculated leaves were sampled on days 0 (about 1 h after spray inoculation), 7, 21, and 35. To avoid the sampling of leaves that were not inoculated, on day 0 leaves were labeled. The samples were weighed and homogenized in 10 mM MgCl_2_ solution by an electric mortar on ice. *S.* Typhimurium 14028s CFUs were quantified by plating a dilution series on XLD agar as described for the quantification of *Salmonella* in soil samples. In parallel, residual undiluted plant leaf homogenates were used for enrichment in BPW and RVS as described above to test for the presence of *Salmonella*. The cell counts of *Salmonella* in the leaves were displayed as log (CFU per g leaf fresh weight). The plants were grown in the greenhouse at 20°C, 16 h light.

### Lettuce Transcriptome Profile

To investigate the response to colonization by *S.* Typhimurium 14028s, lettuce plants were exposed to *Salmonella* under sterile conditions. To this end, lettuce seeds were surface-sterilized by washing 5 times for 1 min with sterile tab water, followed by a sterilization step with 3% NaClO (Carl Roth GmbH + Co. KG) for 4 min and subsequently 4 washing steps with sterile tab water for 1 min. The sterilized seeds were placed on ¼ Murashige and Skoog agar medium (MS, Duchefa Biochemie, Haarlem, The Netherlands) at pH 5.6, including vitamins and 5 g/L sucrose. The seeds were incubated overnight in the dark and subsequently for additional 4 days in a climate chamber at 21°C, 16 h light. At the one-leaf stage, plants were transferred to 5 mL ¼ MS medium in 6-well plates (3 plants per well, in 3 replicates) and further incubated for 24 h. After this pre-adaption, MS medium was inoculated with *S.* Typhimurium 14028s resuspended in 10 mM MgCl_2_ to obtain a final cell count of 10^8^ CFU/mL, 10 mM MgCl_2_ solution was used as negative control. After incubation for 24 h, plants were harvested, immediately frozen in liquid nitrogen, ground with sterile mortar and pestle and stored at -80°C until the RNA extraction.

### *Salmonella* Transcriptome Profile

To determine the transcriptional response of the rifampicin-resistant *S*. Typhimurium 14028s to different plant-related environments, we designed a new experimental approach. *Salmonella* was grown at 37°C for 18–20 h in LB broth with rifampicin (50 mg/L). The stationary phase cells were pelleted at low speed (1,500 × *g*, 10 min), washed twice in 10 mM MgCl_2_ and adjusted to OD_600 nm_ = 1. Two milliliter of this suspension were pipetted into cellulose ester dialysis tubes with a pore size of 100 kDa (Spectrum Europe B.V., Breda, The Netherlands). Closed dialysis tubes were placed in 50 mL screw cap centrifugation tubes containing: (i) 30 mL of a minimal medium (MM); (ii) 30 mL of a lettuce medium (LM); or (iii) 30 mL of lettuce root exudates resuspended in MgCl_2_ (LE). Lettuce root exudates were collected as described previously (Witzel et al., 2017) with some modifications. Briefly, roots of plants were immersed in sterile distilled water for 1 h, and then transferred to fresh bi-distilled water for another 4 h. The exudates from approximately 25 plants were pooled into a single sample. The medium was filtered and concentrated 10-fold by freeze-drying. The lyophilized root exudates were re-suspended in 10 mM MgCl_2_ prior to use. All treatments were performed in triplicates. The MM consisted of 20% M9 salts (Sigma-Aldrich Chemie GmbH, München, Germany; 5× concentrated), 2 mM MgSO_4_ and 1.23 mM glucose in sterile deionized water. The screw cap centrifugation tubes were incubated at 28°C for 24 h while shaking at 180 rpm. Thereafter, 2 × 0.5 mL from each dialysis tube were mixed with RNAprotect (QIAGEN, Hilden, Germany), incubated for 5 min and centrifugated at high speed (4,000 × *g*, 10 min).

### RNA Extraction, Quantitative PCR, Library Preparation, and Sequencing

RNA was extracted from 0.1 g of lettuce leaves using the RNeasy Plant Mini Kit (QIAGEN) following the manufacturer’s instructions. DNA was digested using the PerfeCTa DNase I (Quanta BioSciences, Gaithersburg, MD, United States). For qPCR analysis, RNA was first transcribed to cDNA using the qScript cDNA Synthesis Kit (Quanta BioSciences) following the manufacturer’s instructions. The subsequent library preparation and Illumina HiSeq sequencing were performed at the IIT GmbH, Bielefeld, Germany.

From *S*. Typhimurium 14028s cell pellets obtained as described above, total RNA was extracted using the RNeasy Mini Kit (QIAGEN). The rRNA was removed using the Ribominus Kit (Thermo Fisher Scientific, Darmstadt, Germany) with some modifications concerning the specificity of *Salmonella* 23S rRNA (Mühlig et al., 2014). For fragmentation of the RNA a Covaris S220 Focused-ultrasonicator (Covaris, Woburn, MA, United States) was used (180 s, 175 W peak power, 10% puty, 200 cycles). The fragments were dephosphorylated and phosphorylated using antarctic phosphatase (NEB, Frankfurt, Germany), SUPERase∙ In and T4PNK (Thermo Fisher Scientific) to gain a common phosphorylation state for all fragments. The library preparation was done with the TruSeq Small RNA Library Prep Kit (Illumina, San Diego, CA, United States) according to the manufacturer’s instructions and the SuperScript IV cDNA synthesis Kit (Thermo Fisher Scientific). Illumina HiSeq sequencing was performed at the ZIEL – Institute for Food & Health, Core Facility Microbiome/NGS, Technische Universität München, Freising, Germany.

### Sequence Processing and Statistical Analyses of Lettuce Transcriptome

Fastq files were trimmed using trimmomatic (Bolger et al., 2014) and then mapped to the *L. sativa* L. cultivar Tizian genome (Verwaaijen et al., 2018) using top hat (Kim D. et al., 2013). The resulting BAM files were read into read explorer (Hilker et al., 2016) and read counts were obtained. Kyoto Encyclopedia of Genes and Genomes (KEGG) mappings for transcripts were obtained by aligning translated transcript sequences to the *Arabidopsis thaliana* protein set (TAIR10, November 2010, arabidopsis.org) using a local implementation of BLAST (Altschul et al., 1997). For each sequence the best hit was used for KEGG pathway analysis and only hits with an *E* < 1^-10^ were used. Statistical analysis was carried out in R (R Core Team, 2018). Differentially abundant genes were determined using DESeq2 (Love et al., 2014). Enrichment analysis of Gene Ontology (GO) terms and KEGG pathways was done with R library gage (Luo et al., 2009) and the KEGG pathway gene set for *A. thaliana* was generated using function kegg.gsets. KEGG pathway maps were plotted with R package pathview (Luo and Brouwer, 2013). The raw sequences were deposited in the Sequence Read Archive database (NCBI) with the Bioproject ID PRJNA507508.

### Sequence Processing and Statistical Analyses of *S.* Typhimurium 14028s Transcriptome

The sequence analyses were performed using the Bowtie2 (Version 0.6), cufflinks (Version 2.2.1.0), cuffmerge (Version 2.2.1.0), cuffquant (Version 2.2.1.1) and cuffdiff (Version 2.2.1.5) (Langmead et al., 2009; Trapnell et al., 2010) where the FPKM (Fragments Per Kilobase Million) and the significant differences between the treatments were calculated as false discovery rate (FDR)-adjusted *p*-values of the test statistic (*q*-values). Values differing by more than twofold and *q*-values below 0.05 were considered significantly increased or decreased in gene expression. Not all genes detected in the *S.* Typhimurium 14028s from the lettuce treatments were detected in those from minimal medium and vice versa. These genes were also considered significantly up- and downregulated, respectively. With the web-based tool PANTHER^1^ significant enriched GO terms among the significant upregulated genes were identified. The Euler diagrams were created using R package eulerr (Larsson, 2018). The raw sequences were deposited in the Gene Expression Omnibus database (NCBI) with the accession number GSE123152 (GSM3497440-GSM3497442 for MM, GSM3497446-GSM3497448 for LE, GSM3497452- GSM3497454 for LM).

### Visualization of Plant Colonization

To visualize the colonization patterns of *Salmonella* on lettuce and corn salad, the plants were incubated with GFP-labeled *S.* Typhimurium 14028s and subsequently investigated using the SP8 confocal laser scanning microscope (CLSM, Leica Microsystems, Wetzlar, Germany). Lettuce and corn salad plants were grown under sterile conditions in 6-well plates as described above. The ¼ MS medium in the wells was inoculated with *S.* Typhimurium 14028s GFP cells to obtain a final cell count of 10^8^ CFU/mL. After 1, 3, or 5 days, the leaves and roots were stained with propidium iodide solution (1 μg/mL) for 5 min and subsequently mounted on microscope slides in 4’,6-Diamidin-2-phenylindol (DAPI) solution (10 μg/mL).

To visualize the colonization of leaf surfaces, a contamination *via* irrigation water was simulated. Lettuce and corn salad leaves were spray-inoculated with a 10 mM MgCl_2_ with 0.05% Tween 20 containing *S.* Typhimurium 14028s GFP (10^8^ CFU/mL). After incubation for 6 days at 20°C in the greenhouse, small squares of the leaves were cut with a sterile scalpel and stained as described above with propidium iodide and DAPI.

All observations were performed using: excitation 405 nm, emission 430–480 nm (presented as blue), followed by a second sequential scan of excitation 488, emission 500–550 nm (presented as green) and excitation 561 nm, emission 600–680 nm (presented as red), including autofluorescence of chloroplasts.

## Results

### *Salmonella* Persisted in Agricultural Soils Throughout the Vegetation Period

**FIGURE 1:**
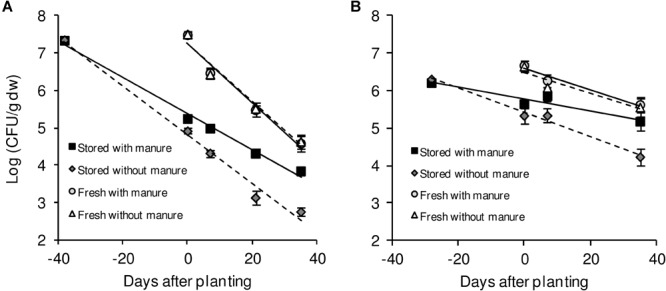
CFU counts of *S.* Typhimurium 14028s in DS and AL soil. Exemplarily shown is the decrease of *S.* Typhimurium 14028s colony forming unit (CFU) counts in diluvial sand (DS) **(A)** and alluvial loam (AL) **(B)** soil treated with or without pig manure. The *Salmonella* strain was introduced together with manure application (stored) or on day 0 directly before planting of lettuce (fresh). Shown are the mean values for treatments and respective standard deviations (*n* = 8). Samples with lettuce and corn salad as crop plants were averaged. Solid and dashed lines indicate linear regressions for soil treated with manure or without manure, respectively.

Contamination with human pathogenic bacteria may occur at all stages of plant production, including the growing period. Since the establishment and persistence of *Salmonella* in agricultural soil(s) are prerequisites of successful plant colonization, we investigated for how long after introduction *Salmonella* would persist in such an environment. Therefore, three different *S. enterica* strains: *S. enterica* serovar Typhimurium strain 14028s (*S.* Typhimurium 14028s), *S.* Typhimurium strain LT2 (*S.* Typhimurium LT2), and *S. enterica* serovar Senftenberg (*S*. Senftenberg) were inoculated into two agricultural soils: diluvial sand (DS) and alluvial loam (AL). Bacteria were inoculated at a cell count of 10^6^ CFU/g of soil. We simulated two different entry routes: (i) *via* organic fertilizer, in this case *Salmonella* was introduced together with chicken litter or pig manure on mixing day, which were selected because both are often contaminated with *Salmonella* and could be potential contamination sources. (ii) *via* irrigation, in this case *Salmonella* was introduced with water on the day of planting, simulating the use of contaminated irrigation water. The CFU counts were monitored during the entire growth period in the different soil conditions. Regardless of the soil type, fertilizer, crop plant or the *Salmonella* strain, *Salmonella* persisted in soil throughout the monitored period, which encompassed the average growth duration for lettuce or corn salad (Figure 1). Even though *Salmonella* was detected in the soil during the entire growth phase, the CFU numbers decreased steadily under all conditions. The results suggest that these human pathogenic bacteria can persist in agricultural soils.

### Soil Type and Fertilizer Application Influenced the Persistence of *Salmonella*

The observed long-term persistence of *S. enterica* in soil motivated us to assess factors with potential impact on the persistence such as: plant species, soil type, fertilizer application and manner of *Salmonella* introduction. In order to compare the changes in abundance of *Salmonella* strains in the different experimental variants, the decrease in CFU was plotted as a function of log CFU/g dry soil and days after inoculation. In the next step, the decrease rates of all experimental variants were calculated and presented as slopes of linear regressions, exemplarily shown in Figure 1 for *S*. Typhimurium 14028s applied to manure-treated or control DS (Figure 1A) and AL (Figure 1B) soils. Based on the slopes of linear regressions, factors influencing the decrease in *Salmonella* CFU were analyzed using a generalized linear model (Table 1). Soil type, fertilizer application and the manner of *Salmonella* introduction significantly influenced the decrease rates (*p* < 0.05), while the plant species did not (*p* = 0.18). Therefore, to improve the readability, we averaged the slopes from lettuce and corn salad samples for each experimental variant (Table 2). The data representing the single plant samples can be found in Supplementary Table S2. The decrease rates of *Salmonella* CFU counts ranged from -0.008 to -0.110 log (CFU/day). Interestingly, decrease rates in DS soil were higher than in AL soil with -0.076 ± 0.014 and -0.034 ± 0.015 log (CFU/day), respectively (Tukey test, *p* < 0.05). A significant interaction was observed in the linear model between fertilizer application and the manner of *Salmonella* introduction. In case of introducing *Salmonella* together with fertilizer, the decrease rates of *Salmonella* were lower compared to the *Salmonella* introduction at transplanting. This indicates a better survival of the strains when introduced to plant production systems *via* organic fertilizers 1 month prior to planting (Table 2). For the tested *Salmonella* strains, the means between the decrease rates were not significantly different (Tukey test, *p* > 0.05) and ranged from -0.048 ± 0.029 (*S.* Typhimurium 14028s) to -0.052 ± 0.020 (*S*. Senftenberg) and -0.064 ± 0.028 (*S.* Typhimurium LT2). In summary, these results revealed that soil type, organic fertilizer and the *Salmonella* inoculation route were major factors influencing the persistence of *S. enterica* in soil.

**Table 1:** Factors influencing the persistence of *Salmonella* in agricultural soils.

Factor	*p*-value
Plant (lettuce, corn salad)	0.1751
Soil type (DS, AL)	<0.0001
Fertilizer (without, pig manure, chicken litter)	0.0016
*Salmonella* inoculation (day 0 or mixing day)	0.0002
Fertilizer application × *Salmonella* inoculation	0.0085

*The decrease in Salmonella CFU counts was analyzed with a generalized linear model based on the slopes of linear regressions. p-values below 0.05 were considered significant.*

**Table 2:** Decline of *Salmonella* in different experimental variants.

			*Salmonella* strains		
Soil type	Inoculation time	Fertilizer	14028s	Senftenberg	LT2
AL	Mixing day (stored)	Without	–0.030	–0.034	–0.052
		Pig manure	–0.016	–0.021	–0.022
		Chicken litter	–0.008	–0.024	–0.018
	Day 0 (fresh)	Without	–0.030	–0.041	–0.063
		Pig manure	–0.028	–0.040	–0.053
		Chicken litter	–0.026	–0.048	–0.052
DS	Mixing day (stored)	Without	–0.088	–0.076	–0.110
		Pig manure	–0.049	–0.053	–0.059
		Chicken litter	–0.074	–0.067	–0.082
	Day 0 (fresh)	Without	–0.073	–0.068	–0.083
		Pig manure	–0.078	–0.073	–0.086
		Chicken litter	–0.080	–0.079	–0.093

*Decrease in log (CFU/g dry weight) counts (n = 4 per time point and experimental variant) of Salmonella strains in different treatments and soil types shown as slopes of linear regressions. Values represent means of slopes from lettuce and corn salad samples for the different treatments in log (CFU/day). For a better readability, the color code indicates a range between high and low decrease in CFU (red and yellow color, respectively).*

### Plant Species and *Salmonella* Strain Determined the Colonization Rate of the Phyllosphere

**FIGURE 2:**
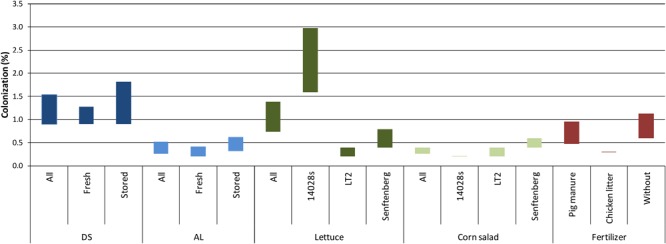
Colonization rates of lettuce and corn salad plants by *Salmonella.* Two plants were pooled into one single 50 mL screw cap centrifugation tube. Only leaves that were not in contact with the soil were sampled. The range of colonized plants (*Salmonella* detected in leaf samples after enrichment) is indicated by colored bars showing the range between the minimal and maximal possible colonization rate. The data are grouped, representing the assessed factors: soil type [diluvial sand (DS)/alluvial loam (AL)], plant (lettuce/corn salad), *Salmonella* strain (14028s/LT2/Senftenberg), time of *Salmonella* application [mixing day (stored)/day zero (fresh)] and fertilizer application (pig manure/chicken litter/without). In total, 3,024 plants were sampled excluding the control plants without *Salmonella* inoculation.

**FIGURE 3:**
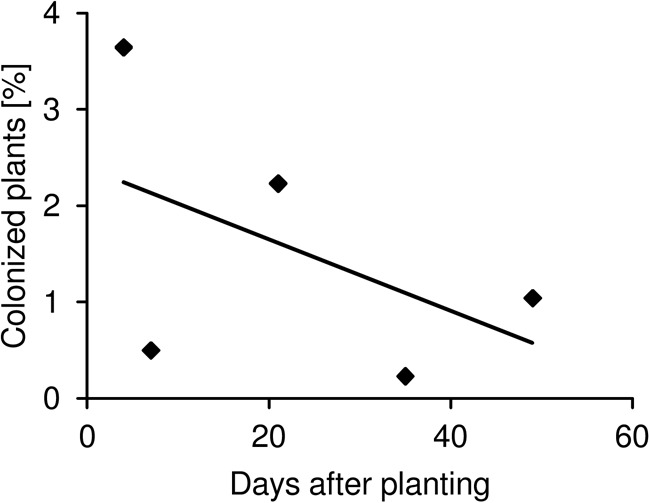
Time-dependent colonization of plants by *Salmonella.* The maximum proportion (maximum range) of lettuce and corn salad plants colonized by *S. enterica* over the experimental period is shown. The plant and soil types as well as the *Salmonella* strains are averaged. The solid line shows the linear regression, which in tendency is decreasing but not significantly (linear model analysis, *p* > 0.05, *R*^2^ = 0.53).

We then asked whether the differences in persistence of *Salmonella* in agricultural soils would influence the colonization rate of crop plants. To answer this question, lettuce and corn salad plants grown under the different conditions described above were harvested during the growth period and analyzed for the presence of *Salmonella* in the phyllosphere. A combination of non-specific enrichment and selective media was used to decide if leaf samples were *Salmonella*-positive. To reduce the number of samples, leaves of two individual plants were pooled into one sample. This resulted in the presented percentage range of plants colonized by *Salmonella* (Figure 2). For a better readability, the data were gathered into groups representing the analyzed factors: soil type, plant species, *Salmonella* strain, manner of *Salmonella* inoculation and the type of organic fertilizer (pig manure, chicken litter, without). In total, the fraction of colonized plants in *Salmonella*-contaminated soil (total sampled plants = 3,024) ranged from 0.5 to 0.9%. In general, a higher percentage of plants was colonized in the *Salmonella*-contaminated DS soil (0.9–1.5%) compared to the AL soil (0.3–0.5%) (Figure 2, Pearson’s Chi-squared test, *p <* 0.05). On average, a higher proportion of lettuce compared to corn salad plants was colonized (0.7–1.4% vs. 0.3–0.4%, respectively, *p* < 0.05 for the maximum values, *p* = 0.12 for the minimum values). *S*. Typhimurium 14028s colonized the highest fraction of lettuce plants (1.6–3%), followed by *S.* Senftenberg (0.4–0.8%) and *S*. Typhimurium LT2 (0.2–0.4%), which displayed the lowest ability to colonize lettuce (*p* < 0.05). For corn salad, no differences between the *Salmonella* strains were observed regarding the number of colonized plants (*p* > 0.05). Furthermore, similar numbers of colonized plants were obtained in the organic fertilizer and control soil treatments (*p* > 0.05). The manner of *Salmonella* introduction (applied with organic fertilizer on day of mixing or on transplanting day zero, about one month after fertilization) did not affect the number of colonized plants in the two soils (Figure 2, *p* > 0.05). Interestingly, the overall colonization rate seemed to decrease over the time of plant growth period. However, this decrease was not significant due to the high variability (Figure 3, *p* > 0.05).

### *S*. Typhimurium 14028s Persisted Better on Lettuce Than on Corn Salad

**FIGURE 4:**
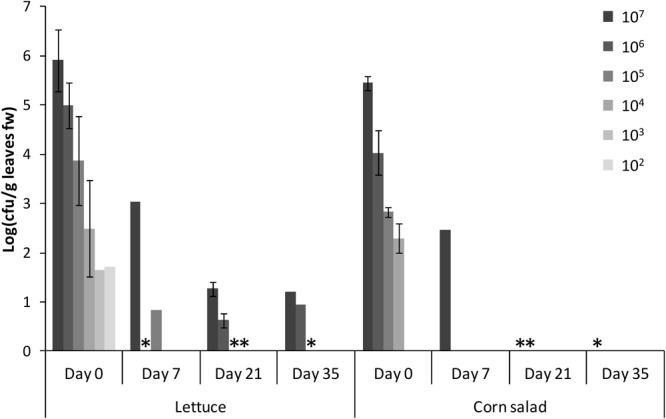
Persistence of *S.* Typhimurium 14028s on lettuce and corn salad leaves. Bars display *Salmonella* CFU counts per g leaf fresh weight (fw) and corresponding standard deviations (*n* = 4) during the days after spray inoculation of the leaves. Asterisks indicate a detection of *Salmonella* only after enrichment. Different cell counts of *Salmonella* (CFU per mL) sprayed on day 0 are indicated by different shades of gray.

One of the biggest differences in the comparisons presented above was the difference in colonization rates between lettuce and corn salad. In order to gain more insights into the ability of *Salmonella* to persist on and in those two crop plants, lettuce and corn salad leaves were spray-inoculated with different cell counts of *S.* Typhimurium 14028s. The presence of *Salmonella* was subsequently determined by CFU counts and in parallel by enrichment and selective plating, during the following 35 days. Generally, directly after spraying (day 0) the CFU of *Salmonella* per g leaf were not different between lettuce and corn salad leaves (Figure 4, *t*-test, *p* > 0.05). Over time, however, the CFU on corn salad leaves decreased much faster. *Salmonella* CFU were still quantifiable on day 35 on lettuce leaves spray-inoculated with the highest two cell counts (10^7^ and 10^6^ CFU/mL) while for corn salad, CFU were quantifiable only until 7 days after spray inoculation. However, after a non-specific enrichment, *Salmonella* was detectable in both plants 35 days post-inoculation. While for corn salad, *Salmonella* was detectable on day 35 only on leaves inoculated with the highest cell count of 10^7^ CFU/mL, *Salmonella* was still detectable on lettuce leaves down to the applied cell count of 10^5^ CFU/mL.

### Colonization Patterns of Lettuce and Corn Salad Plants by *S*. Typhimurium 14028s

**FIGURE 5:**
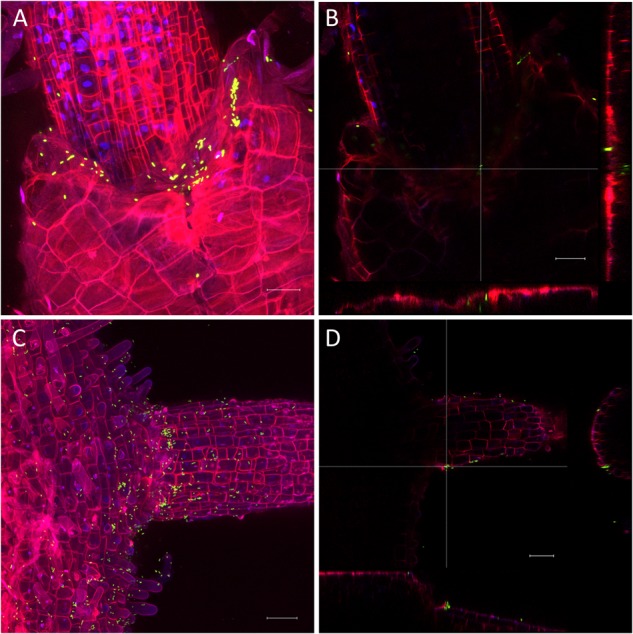
Colonization pattern of *Salmonella* on roots. *S*. Typhimurium 14028s GFP on root of lettuce **(A,B)** and corn salad **(C,D)** grown under sterile conditions. Plants were inoculated with GFP-expressing *Salmonella* for 3 days. Maximum projection images **(A,C)** and orthogonal scalings **(B,D)** indicate nucleus in blue, cell walls in red and GFP-labeled *Salmonella* cells in green. Orthogonal scalings show *S*. Typhimurium 14028s GFP cells in the cavity between primary and secondary root **(B,D)**. The scale bars indicate 30 μm **(A,B)** and 40 μm **(C,D)**.

**FIGURE 6:**
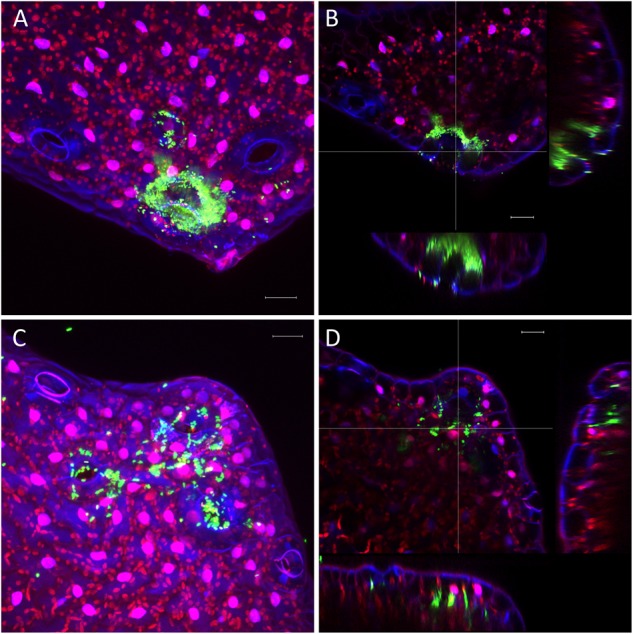
Colonization pattern of *Salmonella* on lettuce leaves. Hydathode regions of lettuce leaves inoculated with *S*. Typhimurium 14028s GFP. Leaves were inoculated with *S*. Typhimurium 14028s GFP for 24 h. Maximum projection images **(A,C)** and orthogonal scalings **(B,D)** show hydathodes, nucleus and cuticula in blue, autofluorescence of chloroplasts in red and GFP-labeled *Salmonella* cells in green. Orthogonal scalings show *S*. Typhimurium 14028s GFP cells inside the hydathode openings **(B,D)**. The scale bars indicate 20 μm.

**FIGURE 7:**
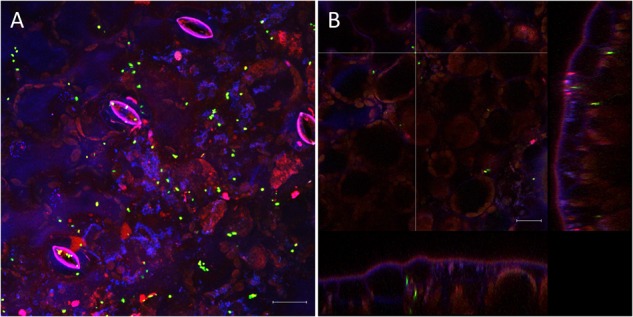
Colonization pattern of *S*. Typhimurium 14028s GFP on corn salad leaves. Representative images were taken 6 days after spray inoculation with *S*. Typhimurium 14028s GFP. Maximum projection image **(A)** and orthogonal scaling **(B)** show stomatal openings and cuticula in blue, autofluorescence of chloroplasts in red and GFP-labeled *Salmonella* cells in green. Orthogonal scaling shows *S*. Typhimurium 14028s GFP cells attached to the cuticula but also inside of spongy parenchyma and stomatal cavities **(B)**. The scale bars indicate 20 μm.

The very diverse results obtained for the persistence on lettuce and corn salad implied that the colonization patterns may differ and that *Salmonella* behaves differently depending on the host plant. To verify this assumption, we assessed the colonization patterns on lettuce and corn salad plants. We used *S.* Typhimurium 14028s expressing the green fluorescent protein (GFP) and a CLSM approach. Two different inoculation methods were used: either plants grown under sterile conditions were inoculated with *Salmonella* in 6-well plates, or plants grown in soil were sprayed with *Salmonella*. In the first approach, the colonization patterns on roots were assessed. Roots of both plants were colonized in a similar manner (Figure 5). Very striking was the augmented colonization in the cavities between primary and the emerging secondary root (Figure 5A,C). *Salmonella* was also detected in the rhizoplane. However, this technique did not allow the detection of *Salmonella* in the deeper root tissues. Regarding lettuce leaves, the hydathodes showed a high abundance of *Salmonella*, also the space in the cavity below the stomata-like opening was frequently colonized (Figure 6). Interestingly, not all hydathode openings were equally colonized, indicating differences in their physiological activity. Leaves of corn salad plants were colonized in a more uniform pattern. *Salmonella* cells were detected attached to the cuticula and also inside the leaf tissue (spongy parenchyma) as well as in the stomatal cavities (Figure 7).

### *S*. Typhimurium 14028s Induced the Defense Response in Lettuce

The very close association between lettuce and *Salmonella* made us wonder how the plant perceives the bacteria. In order to assess how lettuce responses to colonization with *Salmonella*, the transcriptome of whole lettuce plants inoculated with *S.* Typhimurium 14028s was compared to plants exposed to MgCl_2_ (control). Data were obtained from Illumina HiSeq-sequencing of three independent control samples and three independent inoculated plant samples, each comprising three individual plants 24 h after inoculation with *Salmonella*. We obtained approx. 1.23 × 10^8^ raw reads per sample, of which 93.85% passed quality control. Of the quality-filtered reads, 64.27% were uniquely mapped to the genome of lettuce (*Lactuca sativa* L. cv. Tizian), of which 49.05% could be assigned to a feature. The expression of 1,722 gene orthologs was significantly upregulated in the *Salmonella-*exposed plants, if compared to the MgCl_2_ control, while the expression of 1,103 gene orthologs was significantly downregulated (*p* < 0.05, Supplementary Table S3).

**FIGURE 8:**
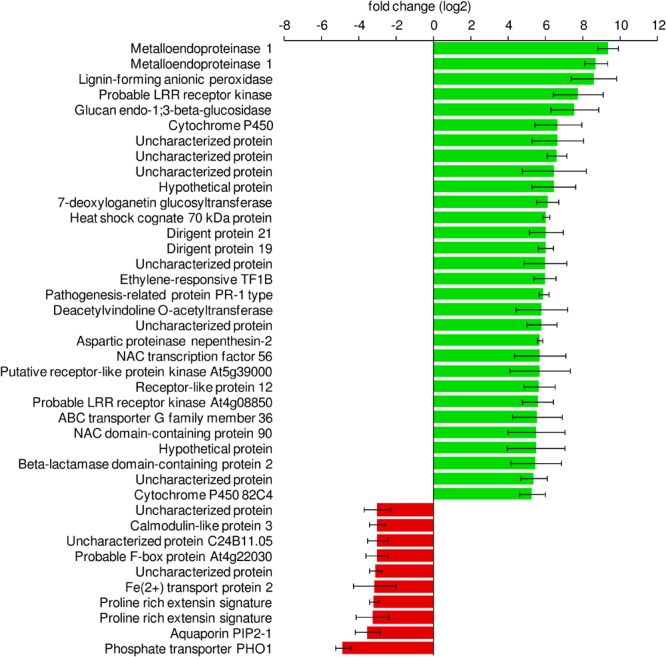
Differentially expressed lettuce genes in response to *S.* Typhimurium 14028s. Sterile lettuce plants were exposed to *S.* Typhimurium 14028s or 10 mM MgCl_2_ for 24 h. The 30 genes with the highest increase in fold change and the 10 with the highest decrease in fold change are shown. Standard errors are indicated by error bars (*p* < 0.05, *n* = 3).

**FIGURE 9:**
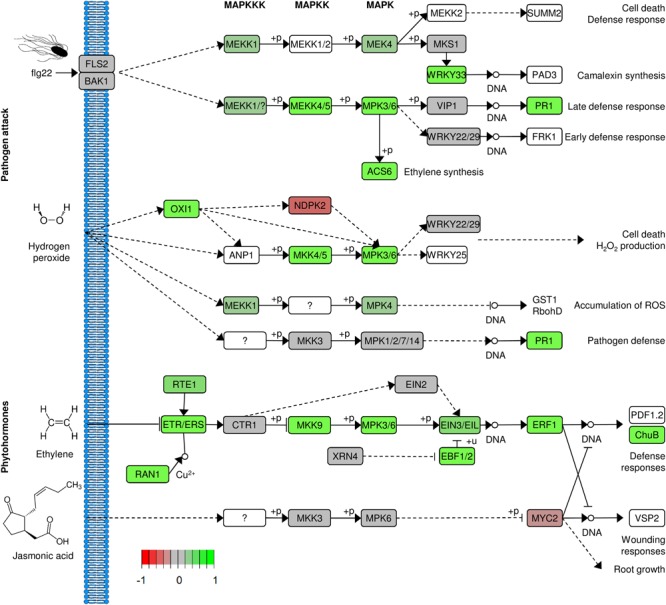
Lettuce response to *S*. Typhimurium 14028s. Changes in signaling pathways related to plant defense response after 24 h exposure to *S.* Typhimurium 14028s, compared to exposure to 10 mM MgCl_2_. Genes were translated to amino acid sequences and those were assigned to Arabidopsis protein orthologs. Up- and down regulated genes in the particular pathways are indicated in green and red, respectively. White background indicates genes not found in the analysis. The figure was based on KEGG and Pathview.

Among the genes with the highest increase in their expression levels after exposure to *Salmonella*, we detected several genes related to defense mechanisms, those genes code for Metalloendoproteinase 1, Pathogenesis-Related Protein PR-1, Glucan Endo-1,3-Beta-Glucosidase and Ethylene-Responsive Transcription Factor 1B. Functional analysis of the differentially expressed genes confirmed that defense-related GO terms were enriched in the group of upregulated genes. Regulation of salicylic acid (SA) biosynthesis as well as the synthesis of lignan and lignin, oxylipins was enriched more than 10 times. Other functional groups included response to stress-related hormones [SA, jasmonic acid (JA), ethylene], defense response or signaling pathways (Figure 8). In-depth analysis of the upregulated genes revealed the presence of multiple very prominent defense-related genes. Several receptors (*FLS2, SOBI1, CERK1, CRK2* and *19, RLK1, RPK2, PK1, RLP2*, and *FER* (*feronia*)), numerous transcription factors (*WRKY19, 23, 30, 33, 40, 41, 50, 55, 58, 70, 71, 75*) together with *MYB4, 44* and *108* and *BHLH61* were among them. Additionally, included were multiple kinases: *MPK3, MKK5, YDA, WAK2, 3, WNK 11* and *WAKL 2, 3, 9, 14* and *15*. Furthermore, several ethylene-related genes (*ERF1A, 1B, 2, 4, 5, 9, 37, 98*, and *110* as well as *ERS1* and *EBF2*) and ubiquitin-related (*PUB17, 22, 23*, and *PP2B13*) genes were upregulated in response to *Salmonella*, all demonstrating that lettuce responded to the colonization with *Salmonella* with a very complex immune response. Water homeostasis- and ion transport-related genes were downregulated in our system. Among them were such prominent genes as the phosphate transporter *PHO1* and *IRT2.* A selection of defense-related GO categories is shown in Figure 9. A full list of enriched GO terms can be found in Supplementary Table S4.

### *Salmonella* Adapted to the Plant Host Nutrients and Adjusted Its Physiology

**FIGURE 10:**
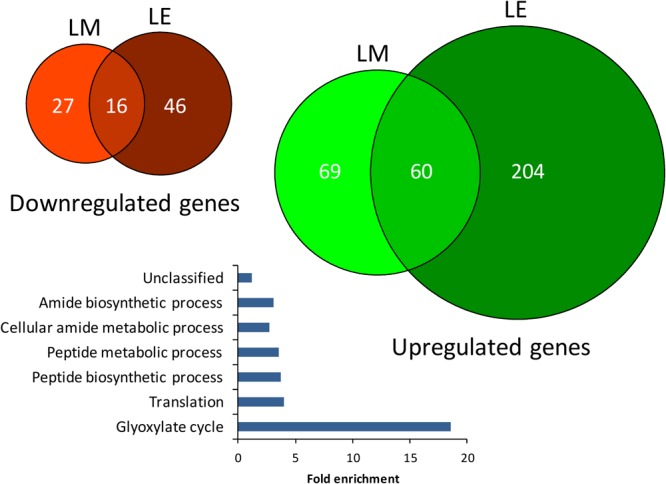
Euler diagram of differentially expressed *Salmonella* genes in lettuce medium and lettuce exudates. The up- and downregulated genes from *S.* Typhimurium 14028s exposed to lettuce-based medium (LM) and lettuce exudates (LE) compared to the minimal medium (MM) as control are shown in green and red, respectively. Indicated are genes with significantly higher or lower expression (*p* < 0.05) as well as genes for which the expression was not detected in the treatment or the control. The bar plot indicates significantly enriched GO-terms of *Salmonella* from genes that were differentially regulated in expression after exposure to LE compared to MM as control.

In a complementary step, we assessed the *S.* Typhimurium 14028s response to lettuce-based medium (LM) or lettuce root exudates (LE). Minimal medium (MM) was used as control. In total, expression of 129 or 264 genes was upregulated in *Salmonella* exposed to LM or LE, while 43 and 62 genes were significantly downregulated, respectively (Figure 10). The complete list of differentially expressed genes including the respective fold-changes and *p*-values is provided in Supplementary Tables S5, S6. GO term enrichment analysis^2^ of all differentially expressed genes revealed six GO terms including, glyoxylate cycle, translation, peptide biosynthetic process, peptide metabolic process, cellular amide metabolic process and amide biosynthesis enriched among genes upregulated in response to LE (Figure 10). Surprisingly, no overrepresentation of functional categories was found in response to LM or in the downregulated genes.

In *Salmonella* exposed to LE, genes associated with amino acid biosynthesis showed the highest upregulation, if compared to MM. Among the genes with the highest increase in expression were *amtB, asnA*, and *asnB* as well as *gltI*, associated with ammonium transport, L-asparagine biosynthesis and glutamate/aspartate transport, respectively. Expression of *cysK* coding for a cysteine synthase A was increased in response to LE, if compared to response to LM. Expression of genes involved in isoleucine synthesis was upregulated in bacteria exposed to the root exudates (*ilvE, ilvA, ilvD, ilvL, ilvJ*) or in both LE and LM (*ilvG*), if compared to MM. The expression of *proP* was higher in LE, compared to LM. The *proP* gene is involved in proline and/or betaine transport and osmoregulation (Culham et al., 1993) and supported the colonization of alfalfa seedlings by *Salmonella* (Kwan et al., 2018). Gene expression of *argR*, which likely plays a role in the control of arginine biosynthesis and aerobic catabolism (Park et al., 1997), was increased in response to LE. Also expression of *ilvG* and *ilvE*, which are part of the ilvGEDAYC cluster encoding enzymes responsible for isoleucine and valine biosynthesis (Blazey and Burns, 1982) was upregulated. Interestingly, expression of *metW, metY*, and *metZ*, associated with methionine biosynthesis (Alaminos and Ramos, 2001; Hwang et al., 2002) was decreased in *Salmonella* exposed to root exudates. Furthermore, expression of several genes associated with *Salmonella* virulence was upregulated, including sulfurtransferase encoding gene *glpE* (compared to LM) and the cytoplasmatic membrane protein encoding gene *dsbB* (in LE compared to MM). Also, expression of *fdnI* and *ogt* was upregulated in response to LE (compared to LM), both were associated with the ability of *Salmonella* to invade host epithelial cells (Lee et al., 2018). Biofilm and curli-associated genes *luxS* and *csgB* were highly expressed in response to LE (compared to MM and LM, respectively).

In contrast to the clear and specific response to LE, *Salmonella* exposed to LM regulates genes associated with a rather general stress response. Among genes with the highest upregulation were *ibpA, clpB, dnaK*, and *dnaJ*. The first encodes a small heat shock protein, reported to stabilize and protect proteins from denaturation and proteolysis during heat and oxidative stresses. Proteins bound to the IbpAB complex were efficiently refolded and reactivated by ClpB, DnaK, and DnaJ, representing ATP-dependent chaperone systems (Melkina et al., 2011). Besides the stress response genes, expression of amino acid biosynthesis genes was increased, e.g., *asnA*, associated with L-asparagine biosynthesis. Furthermore, expression of *feoA* and *feoB*, which code for ferrous iron uptake transporter proteins A and B, was upregulated. Additionally, expression of genes associated with infection and host-pathogen interaction was increased (*feoB*, STM1808, STM0082), as well as genes associated with biofilm formation (*yaiC, luxS*). Interestingly, *sulA*, which is associated with filamentation and downregulation of SPI-1 as well as *celC*, encoding a protein able to degrade cellulose-type substrates, were highly expressed in response to both LM and LE.

## Discussion

### Persistence of *Salmonella* in Soil Is Influenced by the Soil Type and Application of Organic Fertilizer

Untreated livestock waste used as soil fertilizer has been already postulated as a potential route of contamination with *Salmonella* and other human pathogens (Beuchat, 2002). Pre-harvest contamination is of particular concern, since *Salmonella* can internalize into plant tissue and therefore limit the efficacy of post-harvest sanitizers (Solomon and Sharma, 2009). One of the factors influencing the extent of internalization might be the actual cell count of *Salmonella* in soil (Cooley et al., 2003; Hirneisen et al., 2012). Therefore, the identification and understanding of factors that reduce the persistence of *S. enterica* in soil would help to lower the risk of leafy green contamination. Consequently, the fate of *S. enterica* in the manure-amended soil-plant ecosystem and influencing factors are subject to intensive research (reviewed in Ongeng et al., 2015). The persistence of *Salmonella* was better in AL (loamy) than in DS (sandy) soil, which might be connected to the higher nitrogen and organic carbon contents in AL soil and the higher content of clay (Rühlmann and Ruppel, 2005), which is known for its positive effect on retention of organic and anorganic compounds (Basak et al., 2012). In contrast, a previous study indicated that the number of cultivable *S*. Typhimurium LT2 decreased faster in soil treated with sewage sludge (Fornefeld et al., 2018). The authors suggested that stress imposed by the sewage sludge forced *Salmonella* to enter the viable but non-culturable (VBNC) state. Our results support therefore the hypothesis that one of the main factors driving the persistence of *Salmonella* in soil might be the availability and composition of nutrients. The usage of high quality manure (e.g., high in organic matter, low in easily available nutrients) might reduce the persistence of *Salmonella* in soil, as previously suggested by Franz and van Bruggen (2008) for *E. coli* O157:H7. Another cause for the better survival of *S. enterica* in AL compared to DS soil might be the different soil bacterial community structure (Schreiter et al., 2014). Thus, the survival of *S.* Dublin in soils with different soil type and management regime was reported to be correlated with differences in microbial communities rather than with differences in physicochemical properties (Moynihan et al., 2015).

### Root Internalization and Colonization of Plant Leaves

Internalization of *S*. Typhimurium LT2, *S*. Typhimurium ATCC14028, and *S*. Typhimurium S1 into the root of different plants, including lettuce, was demonstrated recently using a sterile system and subsequent FISH-CLSM analysis (Kljujev et al., 2018). In the case of basil, internalization of *S.* Thompson strain FMFP 899 *via* roots was demonstrated using *Salmonella*-soaked germination discs using high cell counts of *Salmonella* for inoculation (Li and Uyttendaele, 2018). In the present study, we could observe all tested *Salmonella* strains in lettuce and corn salad leaves, indicating that the bacteria were able to internalize into the roots and spread within the plant, probably through the vascular system. Previous studies suggested that *Salmonella* preferentially colonizes emerging zones of secondary roots, rather than root hairs or the root surface. This was confirmed in this study by CLSM. Lateral root cracks were suggested to be sources of nutrients and potential entry sites of *Salmonella* and other bacteria into the plant (Dong et al., 2003). However, these internalization events were rather rare, with an average of 0.7–1.4% of positive plants for lettuce and 0.3–0.4% of positive plants for corn salad. The low rate of internalization might be the reason for the scarcity of internalization-related quantitative data in the literature, suggesting that experiments designed for small numbers of investigated plants likely resulted in (false) negative observations. A comparable range of 2.9% *Salmonella*-colonized plants (1 out of 35) was observed for lettuce after surface sterilization (Honjoh et al., 2014).

Interestingly, although we have shown that organic fertilizers can increase the survival of *S. enterica* in soil, the application of pig manure and chicken litter did not affect the colonization rate of the lettuce or corn salad. This is in line with a previous study, showing that sewage sludge amendment does not promote the internalization of *S*. Typhimurium LT2 into lettuce plants (Fornefeld et al., 2018). Nonetheless, we cannot exclude that the increase in *Salmonella* persistence after fertilizer application will ultimately result in a slightly higher colonization rate. Further experiments are required to precisely answer this question.

Intriguingly, a higher proportion of plants were colonized if the plants were grown in DS soil. Although this seems to be in contrast to the better survival of *Salmonella* in AL, a possible explanation might be different bacterial motility, affected by soil physicochemical properties. In this study, the high clay content in AL soil provided a large surface for microbial attachment and might have led to a decreased mobility of *Salmonella*, which eventually led to a reduced exposure of plant roots and finally resulted in lower colonization rates. Additionally, clay minerals are small, generating a large surface area with combined hydrophilic and hydrophobic properties that can provide adsorbed nutrients to other microorganisms (Cuadros, 2017). Differences in the colonization rate between DS and AL soils might be related to differences in the microbial communities. For example, Klerks et al. (2007) reported that internalization was more frequent in axenically grown plants than in soil-grown plants. This was associated with reduced competition between the bacterial community and *Salmonella* and the resulting fitness advantage.

Strikingly, our results from the spray inoculation experiment showed that *S*. Typhimurium 14028s was more persistent on lettuce than on corn salad leaves. This might be caused by the differences in surface properties, leaf structures, microclimates and nutrient availability on the leaves. For example, in the case of tomato, plant surface compounds and exudates had a significant effect on *S. enterica* growth and colonization efficiency (Han and Micallef, 2016). Also the hydrophobic properties of the epicuticular wax layer appeared to be important for the colonization of lettuce leaves by *S*. Senftenberg (Hunter et al., 2015). Additionally, the better persistence on lettuce than on corn salad plants might have been associated with differences in the plant defense system. For example, the number of infiltrated *E. coli* O157:H7 isolate Sakai in lettuce and spinach leaves did not increase over time, indicating an arrested/restricted bacterial growth (Wright et al., 2017). Nonetheless, in the same study the authors observed an increase in bacterial number of >400-fold after infiltration of *Nicotiana benthamiana* leaves, suggesting that this difference was due to differences in the plant defense system.

### Induction of Defense Response in Response to *S*. Typhimurium 14028s

Lettuce response to *Salmonella* colonization seems very clear. The upregulation of multiple defense-related genes indicated that as in case of e.g., *A. thaliana* (Schikora et al., 2011), lettuce perceives *Salmonella* and induces its immune system. Numerous upregulated genes point to rather typical Pattern-Triggered Immunity (PTI) response, among them were receptors which recognize such Pathogen-Associated Molecular Patterns (PAMPs) (*FLS2* and *CERK1*), also the induced expression of several MAP kinases and WRKY transcription factors indicated the ability to perceive the bacteria and to react. Very interesting was the fact that in addition to the upregulated expression of many genes involved in the early response to bacterial invader and signaling cascades, also genes associated with the remodeling of the cell wall were upregulated. Several lignin synthases and enzymes involved in lignin synthesis were among them. In summary, the observed response is very similar to an immune response to typical plant pathogens. Interestingly, a recent report suggested that growth of *S.* Typhimurium 14028s is not affected by the *FLS2*-mediated plant defense in *Medicago truncatula.* At the same time, the growth of *S*. Typhimurium strain LT2 was reduced on the wild-type plant but not on the *FLS2*-deficient mutant (Wahlig et al., 2019). The authors hypothesized that these differences were associated with differences in sensitivity toward reactive oxygen species caused by the defect of *rpoS* in strain LT2. This difference might be one possible explanation for the observed lower colonization rate of lettuce by *S*. Typhimurium LT2 compared to *S*. Typhimurium 14028s.

### Transcriptional Changes of*S.* Typhimurium 14028s in Response toPlant Extracts and Root Exudates

From the three tested *S. enterica* strains, *S*. Typhimurium 14028s was the most successful in colonizing lettuce plants, indicating its ability to adapt to the conditions of lettuce rhizosphere and plant tissues. To assess this potential adaptation, we exposed *S.* Typhimurium 14028s to lettuce root exudates (LE) or lettuce-based medium (LM). Analysis of the transcriptome data showed enrichments in GO terms associated with glyoxylate cycle as well as other metabolic and biosynthetic processes in response to root exudates. The glyoxylate cycle was reported to be upregulated under conditions of oxidative stress but plays also an important role in pathogenesis (Ahn et al., 2016). Furthermore, genes associated with *Salmonella* virulence and biofilm formation were upregulated. This is in line with previous observations, showing that *S*. Typhimurium reacted to root exudates with the induction of pathogenicity-related genes (Klerks et al., 2007). Our study revealed the upregulation of *glpE*, which codes for a sulfurtransferase that was shown to contribute to virulence of *S.* Typhimurium in the murine model (Wallrodt et al., 2013). The expression of *dsbB* was also induced in response to LE. This cytoplasmic membrane protein plays a role in SPI-1 regulation and functions by reoxidizing the DsbA protein. DsbA is required for full virulence in a number of pathogenic organisms, such as *Vibrio cholera, E. coli* and *Yersinia pseudotuberculosis*, for the proper function of their type III secretion system (T3SS) (Lin et al., 2008). Furthermore, DsbA is required for the proper folding of SpiA of the SPI-2-encoded T3SS (Miki et al., 2004). Interestingly, the quorum sensing-related *luxS* gene was induced in response to both LE and LM. LuxS was previously shown to play an indirect regulatory role in biofilm formation (Ju et al., 2018). Additionally, the expression of the minor curli subunit gene *csgB* was upregulated in response to LE, it mediates the formation of curli by the conversion of CsgA from a soluble protein to an insoluble fiber (Hammer et al., 2007) and was reported to be regulated by a sigma subunit of RNA polymerase, σ^S^ (RpoS) encoded by the *rpoS* gene (Robbe-Saule et al., 2006), which was also upregulated in response to LE. Curli assembly is important for attachment, the first step in biofilm formation. Interestingly, in the LT2 strain the *rpoS* gene has a defect, which might explain the observed lower colonization rate of lettuce. Another GO term complex of upregulated genes in response to LE, was associated with amino acid biosynthesis. Since root exudates are sugar-enriched but amino acid-limited, this finding was less surprising (Kwan et al., 2018). Among them, *cysE*, involved in cysteine synthesis, was shown to be required for colonization of alfalfa seedlings (Kwan et al., 2018). The observed induced expression in response to LE indicates that this gene might be also important for the colonization of lettuce roots.

The lettuce-based medium (LM) used in this study simulates rather the conditions within the plant or in damaged plant tissues. Here, a high proportion of genes associated with stress response followed by amino acid biosynthesis, was upregulated. Furthermore, the expression of *feoA* and *feoB* was significantly increased, both code for ferrous iron uptake transporter proteins A and B and play an important role in *Salmonella* pathogenesis (Kim H. et al., 2013). Further genes upregulated in response to LM, were: STM1808, which was reported to support the growth of *Salmonella* during systemic infection of mice (Karlinsey et al., 2012) and STM0082, also referred to as gene *srfN*, which was reported for *S. enterica* to be important for intra-host fitness even though, the exact function of this gene is not known so far (Osborne et al., 2009). Strikingly, in *Salmonella* exposed to root exudates the expression of this latter gene was below detection limit. Expression of *sulA*, known to induce the filamentation in *S.* Typhimurium, preventing the invasion of epithelial cells and also associated with a downregulation of SPI-1-related gene expression (Humphrey et al., 2011), was induced upon the exposure to lettuce-based medium. Similar to the response to root exudates, genes associated with biofilm formation were upregulated also in response to LM. Among them was *yaiC*, also referred to as *adrA*, encoding a GGDEF protein, involved in stimulation of cellulose production (Brombacher et al., 2006), one of the major constituents of the *Salmonella* biofilm matrix (Da Re and Ghigo, 2006). The induced expression of the quorum sensing-related *luxS*, which may (indirectly) play a regulatory role in biofilm formation (Ju et al., 2018) was already mentioned above. Very important seems the upregulated expression of *celC.* Its expression was induced in response to both LM and LE. The gene *celC* encodes a protein able to degrade cellulose-type substrates (Yoo et al., 2004) and therefore might play a role in biofilm formation and even the degradation of plant cell walls (Yaronand and Romling, 2014).

In summary, the transcriptome analysis revealed that *S.* Typhimurium 14028s responded to plant compounds by adapting its metabolism. Furthermore, genes associated with virulence, pathogen-host-interactions and biofilm formation were induced, providing evidence that plants are recognized and used by *Salmonella* as alternative hosts. Interestingly, the adaptations to LE and LM on transcriptome level were quite distinct, suggesting that *Salmonella* distinguishes between the conditions that prevail in the rhizosphere and inside plant tissues. This might be crucial for the colonization efficiency and persistence.

## Conclusion

The *S. enterica* strains used in this study were able to survive in soils for several weeks, longer in loamy than in sandy soil and when applied together with the organic fertilizer. Both, lettuce and corn salad were colonized by *S. enterica*, providing evidence for the internalization from the soil *via* the root system. Colonization rates were affected by soil type, plant species and *S. enterica* strain, while lettuce was more frequently colonized than corn salad and plants grown in the sandy soil were more often colonized than plants grown in the loamy soil. *S.* Typhimurium 14028s responded to lettuce medium and lettuce root exudate medium by upregulation of genes associated with metabolism, stress response as well as biofilm formation and virulence, indicating that plants are recognized and used by *Salmonella* as alternative hosts. Also, lettuce reacted to *S.* Typhimurium 14028s with strong upregulation of genes associated with plant immune response, indicating that as in case of Arabidopsis, plants perceive *Salmonella* very similar to typical plant pathogens. Taken together, our results reveal that successful strategies for prevention of food-associated disease outbreaks will need to regard the plant production environment as a whole system, including the soil type, the fertilization management practice and maybe most importantly the crop plant.

## Author Contributions

SJ, JS, RG, KS, and AS were responsible for research design and concept and wrote the manuscript. SJ, JS, MB, and AS performed the experiments and laboratory work. BF processed the plant transcriptome sequences. SJ and JS processed the *Salmonella* transcriptome sequences. SJ, JS, and AS analyzed the data.

## Conflict of Interest Statement

The authors declare that the research was conducted in the absence of any commercial or financial relationships that could be construed as a potential conflict of interest.

## Footnotes

1 http://geneontology.org

2 http://www.geneontology.org
